# “Trouble switching off”: negotiating sleep, screens, and digital device use among young adults

**DOI:** 10.3389/fpubh.2026.1803492

**Published:** 2026-04-24

**Authors:** Yuanbo Qi

**Affiliations:** Department of Communication Studies, College of Humanities, Donghua University, Shanghai, China

**Keywords:** bedtime digital device use, knowledge-behavior gap, psychological self-care, screen use and sleep quality, sleep routines, young adults

## Abstract

**Introduction:**

The paradoxical nature of the widespread phenomenon of digital device use during nightly routines before sleep is intriguing. However, despite the widespread recognition of medical knowledge that blue light may induce sleep disturbances, numerous individuals persist in this behavior.

**Methods:**

This study examines how young adults use digital devices before sleeping, drawing on focus groups from 75 participants to uncover individuals' motives, behaviors, and subjective rationales.

**Results and discussion:**

The study's findings indicate a knowledge-behavior divide, as 79% of participants acknowledged their awareness of potential concerns regarding the use of bedtime devices but continued to engage in this behavior. The most prevalent factors were routine media consumption (80%), psychological needs such as relaxation (40%), distraction from rumination (40%), and bedtime storytelling equivalent (37%). The findings of this study suggest that the use of electronic devices during bedtime is a complex negotiation between psychological self-care and biomedical advice. This reveals the limitations of health informational interventions in addressing culturally normalized behaviors among young adults.

## 1 Introduction

“I know that looking at my phone before sleep is bad—everyone says the blue light harms sleep quality—but when I lie in bed, I just can't relax. My mind keeps running. So I pick up my phone and watch some short videos or scroll through feeds, and strangely, that's what really helps me calm down and finally fall asleep. It has become part of my nightly routine.” (Participant 5, Focus Group 1) (其实我知道睡前看手机不好——大家都说蓝光会影响睡眠质量。但我一躺到床上就很难放松, 脑子一直在转。所以我就拿起手机刷点短视频或者看看动态, 反而这样更容易让我慢慢平静下来, 然后才睡得着。现在这已经变成我每天晚上的一种习惯了).

The quotation provided by one of our participants is not an isolated instance; it is a snapshot of a widespread habitual behavior among young adults who, while reclining in bed, scrolling through social media or listening to podcasts, their faces illuminated by screens until they fall asleep (1, 2). For a significant number of young adults aged 18–35, the pre-sleep transition has become one of their most frequent periods of digital engagement, with electronic devices serving as constant bedtime companions (3, 4). Despite the awareness of medical advice cautioning against nocturnal screen exposure, particularly the disruptive effects on melatonin production and circadian rhythms, young adults routinely persist in these behaviors (5, 6). This paradoxical reliance on digital devices brings up significant concerns regarding the influence of sociocultural norms, psychological motivations, and habitual practices on the bedtime routines of this age cohort.

To contextualize this study within the broader discourse, existing research can be mapped across three distinct domains. First, a robust biomedical consensus establishes that pre-sleep device usage (7–15) causally inflicts cognitive and emotional impairments (16–22). Second, behavioral research highlights a profound knowledge-behavior paradox, demonstrating that young adults persist in these routines despite possessing adequate health awareness (5, 6). Third, theoretical frameworks in behaviorism conceptualize this persistence as experiential avoidance, wherein devices serve as compensatory tools for emotional regulation (23–28). This study locates itself precisely at the intersection of these domains. Because prior quantitative literature hints at these underlying motives (3, 29), this field requires a more qualitative lens to fill a critical gap by exploring how users subjectively rationalize this paradoxical engagement.

Consequently, this study does not seek to measure physiological indicators such as melatonin suppression or brain activity. Rather, it emphasizes the psychological needs, cultural practices, and subjective interpretations of bedtime routines, adopting sociological and behavioral perspectives to comprehend how young individuals framing device use that contradicts medical warnings. The fundamental objective of this study is to understand the rationales that drive this behavior, guided by the following research questions:

RQ1: What subjective rationales and motivations do young adults express for using digital devices before sleep?

RQ2: How do young adults reconcile their awareness of medical advice with their continued bedtime digital device usage?

RQ3: What psychological, habitual, and social mechanisms underlie this behavior?

Ultimately, this study makes certain contributions to existing knowledge. First, this investigation underscores the paradoxical discrepancy between the medical advice and daily practices of young adults in relation to their digital device utilization during the sleep preparation phase. Secondly, it offers a qualitative behavioral perspective on the ways in which digital devices serve as instruments and remedies for emotional regulation, tension relief, psychological routines, and habitual routines at bedtime. Third, it emphasizes the normalization and routinization of this digital phenomenon as a potentially global, sociocultural phenomenon among young adults, rather than as an individual decision. Moreover, it deepens our comprehension of the subjective agency of young adults in framing digital device utilization during their sleep preparation stages as advantageous, despite their awareness of its physiological costs. In addition, it contributes to the existing interdisciplinary comprehension of these issues at the intersections of media studies, sociology, psychology, and human-computer interaction by providing a qualitative, empirically grounded examination of pre-sleep digital media practices. Finally, it offers valuable insights for future interventions in digital wellbeing initiatives and sleep health education by examining the psychological and cultural functions that digital devices fulfill.

### 1.1 Literature review

There is an increasing corpus of literature examining the relationship between bedtime electronic device usage and sleep quality, consistently providing strong evidence for a negative association. Early studies by Exelmans and Van den Bulck (14) identified pervasive mobile device use as a rapidly spreading global health issue. Subsequent research has reinforced this; for instance, a study based in Saudi Arabia by AlShareef (9) and a survey of 396 university students by Pham et al. (7) both demonstrated that pre-bedtime technology use is significantly correlated with reduced sleep duration, diminished sleep satisfaction, and excessive daytime sleepiness (8, 30). This multilevel impact extends across various age demographics, negatively affecting sleep assistance and daytime functionality in preschoolers (13), delaying sleep and disrupting cycles in adolescents (11, 12), and impairing academic performance (10). Ultimately, drawing on large-scale population studies, researchers such as Hysing et al. (15) have advised strict restrictions on electronic devices to mitigate these negative outcomes. While much of this literature relies on correlational data, clinical experimental studies have firmly established a causal relationship between restricted sleep and adverse cognitive and emotional outcomes (18, 19, 21, 22). The dominance of this biomedical framing has effectively established—statistically, experimentally, and empirically—that bedtime device usage is fundamentally harmful to sleep.

These physiological disruptions translate into severe, measurable impairments that extend into both cognitive and emotional domains. Clinical experiments demonstrate that even minor, chronic sleep restriction—common among young adults who delay sleep for screen use—produces cognitive deficits in sustained attention and working memory equivalent to up to two nights of total sleep deprivation (20, 21). Beyond cognitive performance, sleep loss fundamentally alters the brain's emotional circuitry. Neuroimaging research reveals that sleep deprivation physically disrupts the functional connectivity between the prefrontal cortex and the amygdala, resulting in a 60% amplification in emotional reactivity and a marked increase in psychological distress (19, 22). Consequently, the “worse sleep” reported by device users is not merely a matter of tiredness; it is a state of systemic impairment that compromises academic learning, executive decision-making, and long-term psychiatric health (16, 17).

Despite the robust statistical and causal correlations established by quantitative methodologies, critical gaps remain regarding the experiential nuances that explain why young adults continue engaging in bedtime device use despite being fully aware of its harmful effects. Most young adults are well informed of the biomedical warnings against screen usage before bed, yet the practice remains deeply embedded in their daily routines (5, 6). To understand why this persistent knowledge-behavior gap exists across diverse populations through the lens of behaviorism is helpful. Seemingly maladaptive habits are maintained because they serve highly reinforcing psychological functions. Late-night screen use might be understood as a form of experiential avoidance—a deliberate coping strategy to escape uncomfortable private experiences, such as academic anxiety or intrusive thoughts (24). Theoretical models of compensatory internet use and the escape pathway suggest that digital devices function as accessible tools for tension relief (23, 25). In the pre-sleep transition, the smartphone acts as a pacifying technology that provides immediate psychological comfort (26), framing bedtime procrastination as a short-term emotion-regulation strategy in which individuals prioritize immediate mood repair over long-term physiological health (27, 28).

What remains insufficiently explored, however, is precisely how this phenomenon has become psychologically normalized from the users' own subjective perspectives. While previous studies have hinted at possible psychological explanations (3, 29), there has been no systematic qualitative exploration to highlight the nuances of how young adults rationalize and justify their constant bedtime engagement with screens. As such, this study seeks to bridge this gap by adopting a perspective rooted in functional analysis to explore the mental and sociocultural dimensions of bedtime digital device usage. This approach underlines how young adults understand and experience this behavior, highlighting their subjective agency in conceptualizing digital devices as necessary tools for emotional regulation and routine stabilization, despite their awareness of the biological costs.

## 2 Materials and methods

### 2.1 Research design

This study adopts a qualitative research design to investigate how young adults interpret, normalize, and negotiate the use of digital devices during the period immediately preceding sleep. Rather than approaching bedtime screen exposure solely as a physiological or clinical phenomenon, the study examines the social meanings, psychological rationalizations, and habitual practices through which individuals integrate digital media into their nightly routines. The central analytic concern is how participants reconcile widespread knowledge regarding the negative health implications of bedtime screen use, particularly the effects of blue-light exposure and cognitive arousal, with their continued reliance on digital devices as functional tools for relaxation, distraction, emotional regulation, and routine stabilization. These tensions, which are embedded in everyday experience and articulated through narrative reasoning rather than explicit behavioral calculation, cannot be adequately captured through experimental or survey-based approaches alone; instead, they require in-depth qualitative inquiry into subjective interpretation, discursive justification, and social normalization.

Data were collected through a series of in-person focus groups conducted over a four-week period between September and October 2024 on a large metropolitan university campus that served as a neutral meeting site for both students and non-student participants. Although several participants were enrolled in higher education, the study was not framed as an investigation of university populations *per se*. Instead, the sampling strategy targeted a specific young adult demographic, operationalized as individuals aged 18–35 (born approximately between 1989 and 2006, encompassing younger Millennials and Generation Z) who regularly engaged with digital screens during the 2 h preceding sleep. Participants were recruited through campus bulletin advertisements, social media postings, and community mailing lists, allowing individuals from varied occupational backgrounds to participate while preserving a shared generational cohort and technological environment.

The focus-group format was selected because bedtime media practices are often routinized, tacit, and socially negotiated. Group discussion enabled participants to articulate taken-for-granted habits, respond to others' experiences, and collectively reflect on contradictions between medical advice and everyday conduct. These dialogic dynamics were particularly important for capturing the moral reasoning, self-awareness, and ambivalence that structured participants' accounts of screen use at night. Across sessions, participants frequently corrected, extended, or re-evaluated one another's claims, generating rich material on the social circulation of norms surrounding sleep hygiene and digital consumption.

### 2.2 Participants and sampling rationale

A total of 75 participants took part in the study, distributed across seven focus groups containing between 9 and 12 participants each. Eligibility criteria required that participants (1) be between 18 and 35 years of age and (2) report routine engagement with at least one digital device—such as a smartphone, tablet, or laptop—during the 2 h preceding bedtime on most nights. These criteria ensured that the dataset captured individuals for whom bedtime screen use constituted a meaningful and habitual practice rather than an occasional activity.

Sampling was purposive rather than statistically representative. The goal was to ensure diversity in occupational background, educational attainment, residential context, and patterns of screen engagement, thereby maximizing interpretive variation and enabling comparison across different experiential orientations toward nighttime media use. Participants included university students, early-career professionals, and individuals employed in service, creative, and technical occupations, while residential contexts ranged from dense urban centers to suburban areas. Although several participants were affiliated with universities, the study was not designed specifically as a student sample but rather targeted the broader young adults in this cohort within a shared technological environment. Participants were recruited through campus bulletin advertisements, social media announcements, and community mailing lists. Prior to participation, individuals completed a short screening questionnaire collecting basic demographic information including age, occupation, education level, residential environment, and approximate duration of screen use before sleep (See Table 1). These responses were used solely to ensure balanced group composition and to verify that participants met the study's eligibility criteria; they were not treated as variables for statistical analysis.

**Table 1:** Participant demographics.

Demographic category	Group	*N*	%
Gender	Male	37	49.3%
	Female	38	50.7%
Age group	18–24 years	42	56.0%
	25–29 years	20	26.7%
	30–35 years	13	17.3%
Education level	Secondary Education	6	8.0%
	College Diploma	12	16.0%
	Bachelor's Degree	44	58.7%
	Master's Degree or Higher	13	17.3%
Occupational background	University Students	32	42.7%
	Early-Career Professionals	43	57.3%
Residential context	Urban Center	52	69.3%
	Suburban Area	23	30.7%
Bedtime screen engagement duration	0–1 h	18	24.0%
	1–2 h	12	16.0%
	2–3 h	10	13.3%
	3+ h	7	9.3%
	Unclear	28	37.3%
Device dependency (self-reported)	Strong dependency (cannot sleep without device)	45	60.0%
	Moderate dependency (frequent use but optional)	25	33.3%
	Low/No dependency	5	6.7%
Focus group participation	Group 1	10	13.3%
	Group 2	11	14.7%
	Group 3	12	16.0%
	Group 4	11	14.7%
	Group 5	10	13.3%
	Group 6	9	12.0%
	Group 7	12	16.0%

All focus group sessions were facilitated by a single moderator with formal academic training in qualitative research methodology and prior experience conducting focus group research. Using the same facilitator across sessions ensured consistency in questioning style, moderation approach, and group interaction dynamics, while also allowing the moderator to develop familiarity with the conversational patterns emerging across groups. Discussions were guided by a semi-structured discussion protocol designed to initiate reflection on bedtime media routines, emotional states before sleep, awareness of sleep-health advice, motivations for device use, and experiences of attempting to limit nighttime screen engagement. Example prompts included questions such as “What do you typically do with your phone or other devices before falling asleep?” and “Have you ever tried to stop using screens at night, and what happened?” However, these prompts merely provided an initial framework for discussion, participants were encouraged to respond to one another, elaborate freely on their experiences, and introduce additional perspectives, allowing disagreements and reflections to emerge organically through open dialogue.

Data collection took place between September and October 2024. The seven focus group sessions were conducted at intervals across this period, with each session lasting approximately 2–3 h. Sessions typically began with a brief introduction to the research topic, followed by open discussion guided by the semi-structured prompts. As the conversations progressed, the moderator posed follow-up questions in response to participants' comments and reactions, encouraging further clarification, elaboration, and reflection. Participants were also encouraged to respond to and challenge one another's perspectives, allowing the discussion to develop through interactive dialogue. This extended format enabled the conversations to move beyond initial descriptive accounts toward deeper reflection on habitual practices and the perceived contradictions between sleep-health knowledge and everyday behavior. Individual sessions concluded when discussion began to stabilize and no substantially new perspectives were emerging. Across sessions, data collection continued until thematic saturation was reached. All participants provided informed consent prior to participation and agreed to audio recording of the sessions. All procedures were conducted in accordance with relevant ethical guidelines and regulations.

### 2.3 Analytical and coding procedure

All focus group discussions were conducted in Mandarin Chinese. Audio recordings were transcribed verbatim and analyzed using thematic analysis following Braun and Clarke's multi-stage procedure: data familiarization, initial code generation, theme construction, theme review, theme refinement, and final synthesis (31). This approach was selected for its capacity to capture both manifest content and latent interpretive structures within participants' narratives. The analysis focused on how individuals described the functions of bedtime screen use, justified continued engagement despite health warnings, articulated emotional states surrounding sleep, and positioned their practices within broader generational norms. Participant quotations are identified using the format (Participant X, Focus Group Y), where X refers to the participant number within the respective focus group and Y indicates the focus group session. All quotations were translated into English for reporting purposes.

Coding was conducted at the level of individual participants. Single contributions frequently contained multiple codable elements—for example, a participant might simultaneously describe anxiety reduction, habitual reliance, and awareness of physiological harm—so multi-coding was permitted when warranted. All transcripts were imported into NVivo 14 to facilitate systematic comparison across groups and to allow iterative refinement of the coding structure. The coding instrument was developed inductively from repeated patterns observed across the focus groups but was oriented toward capturing psychologically and socially grounded mechanisms rather than biomedical outcomes. Two rounds of codebook revision were conducted to ensure conceptual clarity, parsimony, and analytic saturation. A subset of transcripts was independently coded by another analyst using the finalized coding schema; inter-coder reliability reached a Cohen's κ of 0.81, indicating strong agreement.

The final codebook comprised four overarching thematic domains within which specific codes captured recurrent experiential logics such as relaxation, narrative consumption, distraction from rumination, environmental regulation, dependency, normalization, and the knowledge–behavior gap. These codes were subsequently used to generate prevalence estimates reported in the Results (See Table 2).

**Table 2:** The codebook for analysis of bedtime digital device use.

Theme	Code name	Definition	Criterion	Examples
Psychological mechanisms				
	Relaxation	Use of digital devices to promote calmness and emotional settling prior to sleep.	Participant describes device use as helping them relax or settle emotionally before sleep without explicitly referring to intrusive thoughts or anxiety.	“…watching ‘Plants vs. Zombies' videos has been my guaranteed way to fall asleep… it might be because the projectiles just move across the screen horizontally in such a predictable, rhythmic pattern.”
	Bedtime storytelling	Use of digital media (e.g., audio-visual narratives) as a functional equivalent of childhood bedtime stories, providing comfort and familiarity.	Participant reports viewing familiar content or listening to narratives that replicate the comforting effect of bedtime storytelling, reinforcing a sense of safety and security.	“Honestly, I feel like watching familiar videos at night is like having bedtime stories when we were kids. I have this habit of playing the same kind of relaxing content before bed, and it feels comforting, almost ritualistic…”
	Casual information input	Use of media for leisurely, non-work-related information gathering before sleep, typically associated with hobbies or personal interests.	Participant mentions browsing or consuming content unrelated to professional obligations as part of their pre-sleep routine.	“I usually just scroll through some digital product reviews or look at travel blogs. It has nothing to do with my major, it's just a light hobby to look at before closing my eyes.”
	Cheering-up	Use of digital media to improve mood, provide entertainment, and create a positive emotional state prior to sleep.	Participant describes engaging with uplifting or amusing content to elevate mood and prepare for sleep.	“Sometimes I purposefully save funny pet videos or stand-up comedy clips for the end of the day. It just makes me laugh and puts me in a good mood right before I sleep.”
	Distraction from rumination	Use of media to divert attention from intrusive thoughts, worries, or cognitive overactivity that would otherwise interfere with sleep onset.	Participant explicitly mentions worrying, overthinking, or future concerns and uses device engagement to redirect attention away from those thoughts.	“When I lie down at night, if I don't look at my phone, I keep thinking about things — exams, work, everything coming tomorrow — and then I feel anxious and restless... watching short videos actually helps me forget all that.”
15.6-7.2,-38.7498pt	Surrounding adjustment	Use of devices to modify the immediate physical setting conducive to sleep (e.g., adjusting light, sound, or atmosphere).	Participant describes employing devices to create ambient noise (e.g., white noise, rain sounds) or adjust lighting or other environmental conditions.	“I can't stand complete silence, so I always use an app to play the sound of rain or a fireplace. I just leave the phone on the nightstand to create that atmosphere.”
Habitual and routine practices				
	Routine media use	Regular media consumption immediately prior to sleep as part of an established nightly ritual.	Participant indicates habitual device use before sleep without necessarily articulating a clear reason but perceiving it as reliable for sleep onset.	“I don't even think about it anymore. Brushing my teeth, turning off the light, and then opening TikTok for 20 min—it's just the standard procedure every single night.”
15.6-7.2,-38.7498pt	Device dependency	Expressed reliance on digital devices as a necessary aid for falling asleep, reflecting an inability to sleep without device use.	Participant explicitly states that they cannot fall asleep unless they engage with a digital device prior to or during the attempt to sleep.	“If my phone is dead or not next to my pillow, I genuinely cannot fall asleep. I will literally toss and turn for hours until I can hold it and look at the screen.”
Social and normative factors				
	Social connection	Use of social media or communication applications prior to sleep to maintain social awareness and avoid feelings of disconnection or exclusion.	Participant describes checking messages or social platforms before bed to feel connected or informed about peers' activities, to catch up and be updated.	“I have to check WeChat Moments one last time before sleeping. If I don't, I feel like I might miss out on what my friends are doing or a late-night group chat.”
	Normalized use	Framing digital device use before sleep as a normative, culturally accepted behavior.	Participant portrays bedtime device use as an inevitable or routine part of contemporary life.	“I mean, who doesn't look at their phone in bed nowadays? It would be weirder if someone just lay there doing nothing. It's just how our generation lives.”
Contradictory awareness				
	Knowledge-behavior gap	Participants explicitly recognize that digital device use before sleep may impair sleep quality but nonetheless maintain the practice.	Participant acknowledges awareness of health advice but continues to engage in device use before sleep.	“I know that looking at my phone before sleep is bad—everyone says the blue light harms sleep quality—but when I lie in bed... It has become part of my nightly routine.”
	Exceptions	Refers to participants who explicitly described intentional deviations from their otherwise habitual or problematic bedtime screen use, often framed as temporary, justified, or anomalous instances.	Code when participants mention specific occasions or reasoning for not using screens before bed (e.g., feeling too tired, having no access to a device, being with family).	“Usually, I'm on my iPad every night, but during the week of final exams, I was so physically exhausted that I just passed out the second my head hit the pillow without even touching a screen.”

## 3 Results

The data reveal a pronounced tendency toward habitual engagement: 80% of participants reported both routine media use and device dependency before sleep, indicating that bedtime screen behavior is less sporadic and more structurally embedded in nightly routines. Psychological coping mechanisms also emerged as highly prevalent motivations. Relaxation (40%) and Distraction from Rumination (40%) appeared with equal frequency, suggesting that participants turn to screens not only out of habit but also as tools for emotional regulation. While both themes relate to managing pre-sleep emotional states, the former refers to creating a calming atmosphere or routine, whereas the latter describes circumstance to divert attention away from intrusive thoughts or worries.

Concurrently, the data reveal a striking tension between knowledge and behavior. Although 79% of participants explicitly acknowledged the health risks associated with bedtime screen exposure, only 9% reported actively resisting device use before sleep. This disparity indicates that awareness alone rarely translates into behavioral restraint, highlighting a persistent gap between cognition and practice. By contrast, social motivations appeared comparatively limited: while 71% of participants described bedtime device use as normalized within their peer environment, only 25% referenced direct social interaction as a motivation. Together, these patterns suggest that nighttime media engagement appears less socially driven and more as an individually routinized strategy for managing emotional states before sleep (See Table 3).

**Table 3:** Prevalence of themes and codes based on participant responses (*n* = 75).

Theme	Code	Participants (*n*)	% of participants
Psychological mechanisms			
	Relaxation	30	40%
	Bedtime storytelling	28	37%
	Casual information input	21	28%
	Cheering-up	19	25%
	Distraction from rumination	30	40%
	Surrounding adjustment	10	13%
Habitual and routine practices			
	Routine media use	60	80%
	Device dependency	60	80%
Social and normative factors			
	Social connection	19	25%
	Normalized use	53	71%
Contradictory awareness			
	Knowledge-behavior gap	59	79%
	Exceptions	7	9%

Percentages represent the proportion of participants whose responses were coded under each sub-theme. Multi-coding applies, and totals may exceed 100%.

## 4 Discussion

Our findings highlight a phenomenon that is highly prevalent among young adults, confirming that this issue is widely shared rather than an isolated individual quirk. The most fundamental finding of this study is the pervasive knowledge–behavior gap observed among participants. Although most respondents were clearly aware of the health advice that nighttime digital device use disrupts sleep cycles—primarily through blue-light exposure and the suppression of melatonin production (32, 33)—they nonetheless continued to engage in this behavior as part of a normalized bedtime routine. This paradox is not incidental but appears to be a defining feature of contemporary sleep practices.

• “I'm well aware that using my phone right before bed can harm my sleep, especially the blue light thing. But honestly, I can't sleep without doing it now. My phone is part of how I wind down, and even if I know it might make sleep worse physically, mentally it feels necessary. It's like a trade-off I accept.” (Participant 6, Focus Group 1) (“我很清楚睡前用手机对睡眠不好, 尤其是蓝光这个问题。但说实话, 现在如果不刷一会儿手机我反而睡不着。手机已经变成我放松的一部分了。就算我知道它可能对身体不好, 但在心理上我觉得这是我需要的一种放松方式, 是一种我愿意接受的取舍。”).

The persistence of this knowledge-behavior gap suggests that bedtime digital engagement is governed by functional necessity rather than a deficit in health literacy. For young adults, screen use often serves as a “cognitive off-switch” required to mitigate pervasive environmental stressors, such as high-stakes academic pressure and social anxiety. Because this reliance fulfills a vital psychological function—providing immediate mood repair and emotional stabilization—standard digital hygiene education alone is unlikely to eliminate the behavior if underlying systemic anxieties remain unaddressed. This implies that pre-sleep media use is an adaptive, albeit physiologically costly, response to a high-pressure landscape where the immediate psychological reward of relief outweighs long-term health warnings. As one participant articulated:

• “At its core, this comes down to the immense academic pressure college students face, leaving them with absolutely no breathing room. If you're constantly fixated on tomorrow's exams or major responsibilities, falling asleep becomes much harder… so the need [for digital distraction] is undeniably there.” (Participant 4, Focus Group 3) (“其实这件事情的本质嘛, 还是因为大学生的学习压力太大了, 让他们根本就没有空……如果一直想着明天要考试, 明天有重要的事情就更容易睡不着……因此这个需求端肯定是有的。”).

Consequently, a notable divergence exists between physiological impact and perceived psychological benefit. While sleep science highlights the negative impact of pre-sleep device usage (4, 34), participants described their usage as a functional cognitive intervention to interrupt rumination. The device serves to “force my thoughts to stop,” preventing the mind from dwelling on the “uncertainties of the future” that arise in the absence of external stimuli. This “purification stage” for clearing anxious thoughts suggests that subjective mental ease takes precedence over medical recommendations. One participant explained how short-form media acts as a tool for distraction:

• “When I lie down at night, if I don't look at my phone, I keep thinking about things—exams, work, everything coming tomorrow—and then I feel anxious and restless. Even though I know it's not healthy, watching short videos helps me forget all that. It gives something else to focus on so I can finally drift off. Without that, I'd just be lying there, overthinking.” (Participant 4, Focus Group 2) (“晚上躺在床上的时候, 如果不看手机, 我就会一直想事情——考试、工作、第二天要做什么——越想越焦虑。虽然我知道这样不健康, 但刷点短视频反而能让我把注意力转移开, 慢慢就能睡着。不然我可能会一直躺在那里胡思乱想。”).

Building on this psychological utility, the second major finding concerns the routinization and normalization of digital device usage as a generational behavior pattern. Among younger participants, device usage has shifted from a conscious choice to a normalized expectation, functioning similarly to traditional pre-sleep routines such as bedtime storytelling (35, 36). One participant noted:

• “Honestly, I feel like watching familiar videos at night is like having bedtime stories when we were kids. I have this habit of playing the same kind of relaxing content before bed, and it feels comforting, almost ritualistic. Even if I've seen the videos before, it still makes me feel safe and helps me fall asleep quicker.” (Participant 12, Focus Group 3) (“其实晚上看一些熟悉的视频有点像小时候听睡前故事。我有个习惯, 就是睡前放一些比较放松的内容当背景。哪怕那些视频我已经看过很多次了, 但它还是会让我觉得很安心, 好像是一种固定的仪式一样, 这样我反而更容易睡着。”).

This pattern often transcends narrative content, relying instead on the mechanical and sensory predictability of media. Participants utilized the visual and auditory rhythms of specific digital formats as stabilizing cues to signal the transition to sleep, effectively replacing non-digital rituals: “Ever since my senior year of high school, watching ‘Plants vs. Zombies' videos has been my guaranteed way to fall asleep… it might be because the projectiles just move across the screen horizontally in such a predictable, rhythmic pattern.” (Participant 7, Focus Group 5) (“我从高三到现在了, 只要看那些≪僵尸大战植物≫的游戏视频就能睡着。后来我思考了一下……可能因为那些个子弹就这样过去, 水平地看过去, 很有规律。”).

The third important finding redefines digital devices as explicit mediators of emotional regulation. Participants employ an influx of media to forcibly interrupt spiraling thoughts, utilizing the device as an artificial “off switch” for an overactive mind. The digital medium is sought out precisely because it requires minimal cognitive effort while consuming enough attention to block out reality: “When I can't sleep, I turn to my phone… I use that mindless information to forcibly hit pause on my own thoughts.” (Participant 6, Focus Group 7). Ultimately, this behavior has solidified into a sociocultural habit that defines a generation's relationship with technology and rest:

• “Scrolling before bed has just become completely normalized; almost everyone around me does it. It's no longer a conscious choice, but just a natural part of the day. Even if doctors warn that it ruins our sleep, most of us will keep doing it because, for our generation, it's simply a part of life.” (Participant 7, Focus Group 4) (“睡前刷手机其实已经变得很正常了, 我身边几乎所有人都会这样做。现在这已经不是一个需要思考的行为, 而是每天自然发生的事情。就算医生说这会影响睡眠, 大多数人还是会这么做, 因为对我们来说, 这已经是生活的一部分。”).

The findings from the study have certain implications. First, the high prevalence shown in this phenomenon may reflect the broader experiential background of high-pressure environments. Previous research has identified characteristics such as high neuroticism, low self-esteem, and perfectionism as risk factors associated with experiencing these feelings (37–39). Our results support this argument; this phenomenon among participants may stem from an interplay of those individual types of characteristics (40, 41). Moreover, the absence of significant differences between demographic groups suggests that the underlying psychological mechanism and driving factor is a generalized one rather than something limited to specific variables. This aligns with the understanding that this phenomenon stems from cognitive-affective processes and could occur in any individual given the right circumstances, rather than being strictly tied to biology or demographics. It is also noteworthy that the root causes of such phenomenon may not be purely individual; the concept of institutional cultures and systematic factors instill these feelings by causing individuals to question their competence and belongings (42, 43).

Besides, the findings from this research highlights a clear need for proactive measures in educational and professional settings. Previous studies have linked the phenomenon to negative outcomes such as impaired work performance, lower job satisfaction, and higher burnout (44, 45). People who reported intense levels of the phenomenon often noted associated stress and anxiety, proposing potential influence on their wellbeing (46, 47). A practical implication is the importance of support and intervention programs. Institutions might want to consider workshops, mentorship programs, and counseling services that help young individuals manage and mitigate these feelings (48). Creating an environment that openly acknowledges the phenomenon might be beneficial—for example, mentorship programs where leaders share experiences normalize the feelings and provide coping strategies. As existing studies have noted, there is a call for clinicians to actively assess and address those who are experiencing “trouble switching off” sleeping problems (49–51). Regular check-ins, support groups, and training in cognitive strategies are practical steps. Recognizing this phenomenon as a widespread challenge is the very initial step—important yet the beginning. The practical responses involved in these studies include institutionalized support mechanisms so individuals do not suffer in isolation (52, 53). Organizations could foster a culture that counteracts the internal doubts illuminated by this study.

Building on these institutional responses, it is helpful to recognize that bedtime device use often functions as a deeply embedded, instrumental routine for managing pre-sleep cognitive and emotional states. Consequently, effective interventions could move beyond solely reiterating the statistical or biomedical consequences of screen exposure and instead prioritize the provision of low-friction, functionally equivalent alternatives capable of fulfilling the same psychological functions as the original activity (54, 55). Since young adults facing intense academic or professional pressures often lack the residual mental energy to design and initiate complex new routines, such alternatives could be integrated into existing institutional support mechanisms or wellbeing programs. For example, universities and workplaces might introduce structured wind-down sessions where individuals practice non-visual pre-sleep routines—such as brief mindfulness, reflective journaling, or audio-based relaxation—that replicate the calming effects of digital media. Much like attending a regular gym session, going for a swim, or taking a yoga class, young adults could practice these guided routines until they become an automated, habitual part of their daily lives. By embedding these practical, “low-energy” instruments within institutional initiatives, it is possible to address the persistent “trouble switching off” phenomenon through habitual replacement rather than mere restriction.

Our study aligns with the ongoing debate about this phenomenon. First of all, the measured prevalence in our studies falls within the broad range reported in previous studies, which have noted widely ranging prevalence rates—as low as about 9% to as high as 80% or more—depending on how this phenomenon is defined and measured (56–58). The value observed in our sample is on the higher end of this spectrum, indicating that our particular population captured a substantial portion of individuals experiencing such issues. The high percentage may be a function of the context; for example, our sample consists of highly competitive young individuals under significant pressures. The literature indicates that differences in screening tools and criteria dramatically affect prevalence estimates (59, 60). Our study reinforces this point, with the prevalence rate we found highlighting the importance of measurements.

Another important comparison regards demographic patterns and the general nature of the phenomenon. Earlier characteristics of the literature often portrayed such feelings as disproportionately affecting certain groups (61). For instance, it was initially thought to be most common among high-achieving women (62). Nonetheless, more recent research has challenged this by arguing that men and women are roughly experiencing similar rates of this phenomenon (1, 62). Our findings are consistent with these insights; we observed no gender gap in prevalence, supporting the view that the phenomenon exists in all genders to a similar extent. This convergence helps settle a part of the debate on gender differences, indicating that any perception of one-sided gender prevalence is likely due to biases or the sample disparities.

In addition to gender, our research confirms the phenomenon occurs across different ages and stages, from students to young professionals. Our sample age reinforces the need to characterize it as an experience rather than something confined to certain groups. Our study also establishes dialogue to broaden the debates about how to interpret and classify this phenomenon. There has been an ongoing debate about whether these feelings represent a normative experience or a distinct psychological problem warranting clinical attention. Notably, the phenomenon is not officially recognized as a disorder in diagnostic manuals (63). It does not appear in DSM-5 or ICD-10, which means that there is no formal consensus on its status or treatment in clinical practice (64). This lack of formal recognition is the reason why systematic information on its prevalence and management has lagged behind. Our documentation of its high prevalence adds weight to the argument that, formal diagnosis or not, this issue has real significance and deserves attention. It suggests that even if we might not label it a disorder, the psychological distress and functional impact associated with it are affecting a large number of people. This aligns with the scholarly viewpoint that this phenomenon should be taken seriously by mental health professionals or institutions even in the absence of a diagnostic label (63, 64). Overall, our study aligns with a growing body of literature that recognizes this “trouble switching off” phenomenon as a widespread experience with meaningful consequences rather than a rare individual habit. This enhances the dialogue by highlighting how prevalent and uniform the feelings are across different people, contributing to deeper understanding of the nuances of this phenomenon and calling for addressing it through practical interventions.

## 5 Conclusions

This research explains how the younger generation interacts with digital gadgets before sleeping and how they view the influence on their sleep quality. The study's findings indicate a complicated behavioral phenomenon, as evidenced by the following arguments. First, despite widespread awareness that using digital electronic devices before bedtime reduces sleep quality and duration, participants overwhelmingly view their use of digital devices as psychologically beneficial, providing emotional relaxation, promoting mood improvement, and distracting them from intrusive thoughts that motivate them to engage in such practices. Additionally, nighttime gadget usage has become a routine and normative behavior among this demographic, incorporated into evening rituals and frequently replacing conventional pre-sleep practices. Furthermore, digital gadgets have an important role in emotional regulation in pre-sleep situations, giving users with perceived stress relief, reducing mental tension, and promoting psychological preparedness for sleep. These data imply that bedtime gadget use is self-sustaining not merely as habitual behaviors, but rather as a culturally rooted, psychologically driven approach that mirrors the emotional requirements, media affordances, and current sleep patterns of the younger generation.

This study has certain limitations, which future studies may be able to address. First and foremost, the focus group methodology's qualitative focus allows for in-depth study of members' own experiences and viewpoints. It restricts generalizability beyond the current study, despite the fact that the great bulk of prior research on this subject provides quantitative insights. Second, this study depends on participants' self-perceptions of sleep quality and bedtime practices without using objective physiological measurements like actigraphy or melatonin tests to confirm their stories. Third, this study did not look at mediators other than age, socioeconomic status, or cultural environment that may influence bedtime media practice disparities. Future research might address these limitations by adding a broader variety of demographics in the study and integrating qualitative and quantitative data, such as sleep-tracking devices and experimental verifications of bedtime media electronic device use. Cross-cultural and longitudinal research would give complementary insights into how such habits change over time and across settings, as well as an assessment of how technology design aspects impact users' sleep-related behaviors.

In conclusion, this study offers a more comprehensive and nuanced understanding of bedtime digital device engagement, framing it not merely as a physiological disruption but as a culturally established and psychologically motivated habit. This research highlights how young adults utilize digital media as an essential instrumental strategy for managing the emotional and cognitive demands of high-pressure environments. These findings contribute to a multidisciplinary dialogue across sleep studies, psychology, and human-computer interaction by demonstrating that pre-sleep digital practices fulfill critical functions—such as mood regulation, cognitive decompression, and emotional comfort—that biomedical warnings alone fail to address. Ultimately, this research underscores the necessity of shift in health policy: future interventions must move beyond physiological restriction and instead prioritize the development of functionally equivalent, low-friction alternatives that address the underlying psychological and social drivers of nightly digital reliance.

## Data Availability

The original contributions presented in the study are included in the article/supplementary material, further inquiries can be directed to the corresponding author.
